# Effect of hyperchloremia on mortality of pediatric trauma patients: a retrospective cohort study

**DOI:** 10.1590/1516-3180.2022.0370.R2.010923

**Published:** 2024-03-08

**Authors:** Kübra Çeleğen, Mehmet Çeleğen

**Affiliations:** I MD. Physician, Pediatric Nephrologist in Division of Pediatric Nephrology, Department of Pediatrics, Afyonkarahisar Health Sciences University Faculty of Medicine, Afyonkarahisar, Turkey.; II MD. Physician, Pediatric Intensivist in Division of Pediatric Intensive Care Unit, Department of Pediatrics, Afyonkarahisar Health Sciences University Faculty of Medicine, Afyonkarahisar, Turkey.

**Keywords:** Mortality, Pediatrics, Multiple trauma, Saline solution, hypertonic, Mortalities, Hyperchloremia, Major trauma, Hypertonic fluid

## Abstract

**BACKGROUND:**

Hyperchloremia is often encountered due to the frequent administration of intravenous fluids in critically ill patients with conditions such as shock or hypotension in the pediatric intensive care unit, and high serum levels of chloride are associated with poor clinical outcomes.

**OBJECTIVES:**

This study aimed to determine the association between hyperchloremia and in-hospital mortality in pediatric patients with major trauma.

**DESIGN AND SETTING:**

This retrospective cohort study was conducted at a tertiary university hospital in Turkey.

**METHODS:**

Data were collected between March 2020 and April 2022. Patients aged 1 month to 18 years with major trauma who received intravenous fluids with a concentration > 0.9% sodium chloride were enrolled. Hyperchloremia was defined as a serum chloride level > 110 mmol/L. Clinical and laboratory data were compared between the survivors and nonsurvivors.

**RESULTS:**

The mortality rate was 23% (n = 20). The incidence of hyperchloremia was significantly higher in nonsurvivors than in survivors (P = 0.05). In multivariate logistic analysis, hyperchloremia at 48 h was found to be an independent risk factor for mortality in pediatric patients with major trauma.

**CONCLUSIONS:**

In pediatric patients with major trauma, hyperchloremia at 48-h postadmission was associated with 28-day mortality. This parameter might be a beneficial prognostic indicator.

## INTRODUCTION

Intravenous fluids are commonly used to restore hypovolemia and ensure maintenance fluid for pediatric patients in emergency departments, intensive care units, and operating rooms.^1^ Hypertonic saline is frequently chosen for cerebral antiedema treatment, especially in severely injured patients with trauma.^2^ Familiar alternative fluids are 0.9% sodium chloride (NS), Ringer's lactate solution, and 3% hypertonic saline (HTS).^3^ Although abundant knowledge has been gathered about the advantages of these fluids over time with their widespread use, a small number of adverse effects related to their biochemical composition, osmolarity, and volume overload have been identified. Acid–base disorders, electrolytic disorders (hyperchloremia, hypernatremia), volume overload, and hemodilution of proteins are major characterized side effects of crystalloid solutions.^4,5^ HTS used for cerebral antiedema treatment and the most commonly used resuscitation fluid NS contains supraphysiological concentration of chloride (Cl^−^).^6^ Hyperchloremia in critically ill patients used to be considered harmless evidence; However, cases during the last decade the current animal model studies have shown that hyperchloremia is related to immune system paralysis, several coagulation problems, and pulmonary disorders.^7^ Pediatric patients with major trauma are predisposed to hyperchloremia in the postresuscitation period because they are usually required to be treated with NS during the resuscitation period of shock, and the increase in HTS use may cause a exposure to high amounts of chloride ion and an elevation in the level of serum chloride. An observational study showed that hyperchloremia served as an independent risk factor for hospital mortality in patients with major trauma.^8^ Although many studies have investigated the effects of hyperchloremia on mortality in adult trauma patients, few studies have addressed this in the pediatric age group.

## OBJECTIVE

This study aimed to determine whether serum chloride levels were related to mortality in pediatric patients exposed to major trauma.

## METHODS

### Research type and sampling

This retrospective, observational, single-center study analyzed the association between hyperchloremia and mortality in pediatric patients with major trauma admitted to the pediatric intensive care unit of a tertiary university hospital between March 2020 and April 2022. The study was approved by the faculty ethics committee (date: April 1, 2022; no: 2022/4).

Children aged 1 month to 18 years with major trauma who received intravenous fluids with a concentration of > 0.9% sodium chloride were enrolled. Patients with an injury severity score (ISS) of < 15, receiving dialysis, missing serum chloride records, staying for < 48 h, or with baseline hyperchloremia (chloride > 110 mmol/L) were excluded. At our institution, an intravenous 3% NaCl bolus is administered every 6 h to prevent an increase in intracranial pressure (ICP) for every major trauma patient without measuring the ICP. The cutoff level of hyperchloremia was defined as a serum chloride level > 110 mmol/L. This threshold definition was used based on published literature.^9^ Delta chloride (Δchloride) was described as the difference between the chloride level 48-h postadmission and the baseline level. All reasons for death within 28 days of admission to the pediatric intensive care unit (PICU) admission were defined as hospital mortality.

### Data collection tools

Patients were divided into survivor and nonsurvivor groups based on the outcome. The total fluid balance was calculated by adding all volumes of fluid administered over 48 h. Fluid management involves both the bolus and continuous infusion routes. Pre-PICU fluid management was not included in this study. Age, sex, vital signs on arrival (mean arterial pressure, heart rate, and temperature), peripheral oxygen saturation, the need for mechanical ventilation, length of the intensive care stay, and outcomes were recorded. The ISS was calculated to determine disease severity on admission. Serum levels of chloride, sodium, potassium, and creatinine were recorded upon admission to the PICU and at 48 h. Counts of white blood cells and platelets, level of hemoglobin, alanine aminotransferase (ALT), and aspartate aminotransferase (AST), and the international randomized ratio (INR) were included. Data were acquired from the patient files and an electronic hospital data management system.

### Evaluation of data

Data were analyzed with SPSS Statistics 22 software (IBM Co., Armonk, NY, USA). In the univariate analysis, continuous parameters were described as mean ± standard deviation (SD) for normal distribution, median, and interquartile range (IQR) for skewed distribution and nonparametric data. Categorical parameters were evaluated using the chi-squared or Fisher's exact tests. Student's t-test was performed for normally distributed data, and the Mann-Whitney U test was used for nonparametric parameters. Significant parameters identified in the univariate comparison were then included in a multivariate logistic regression model to describe the independent risk factors for 28-day mortality using the maximum likelihood method and backward stepwise selection. Hosmer–Lemeshow goodness-of-fit was used to determine the logistic regression model fit. Significance was set at P < 0.05 for all results.

## RESULTS

Eighty-six pediatric patients with major trauma (56 boys and 30 girls) that met the inclusion criteria were included: 66 (77%) survived and 20 (23%) died within 28 days of PICU admission. Nine patients with an ISS of < 15, two patients with hyperchloremia at presentation, and two patients who stayed in the PICU for < 48 h were excluded. No significant differences were present between the mechanisms of trauma, traffic accidents (n = 41, 48%), or falls (n = 45, 52%), and no significant differences were found between the trauma types in terms of survival (P = 0.95). The mean age of the survivors was 91.32 ± 54.27 months while the age of nonsurvivors was 96.90 ± 55.49 months (P = 0.77). The main characteristics of the patients with major trauma are shown in Table 1. No significant differences were found between survivors and nonsurvivors for respiratory rate, heart rate, mean arterial blood pressure, peripheral oxygen saturation, or body surface area (BSA). The total infused fluid volume was greater in nonsurvivors than in survivors (5.1(2.8) vs. 5.8(3.7); P = 0.04). The ISS differed significantly between the two groups (P = 0.01). The median duration of mechanical ventilation was significantly longer in nonsurvivors (4(2) vs. 5(3); P = 0.80). No significant difference was present in the mean duration of the total length of PICU stay (8.61 ± 2.60 versus 5.90 ± 2.23; P = 0.09). Initial electrolyte levels were similar in the two groups; however, the 48-h sodium level was significantly higher in nonsurvivors (149.9 ± 6.88 versus 142.67 ± 2.62 mmol/L, P ≤ 0.01). Chloride levels > 110 ng/mL were significantly higher in nonsurvivors at 48 h (P = 0.05). Patients in the nonsurvivor group did not have significantly higher serum chloride levels on admission compared with survivors although these levels in nonsurvivors were significantly higher at 48 h (103.76 ± 2.52 versus 104.60 ± 2.83 mmol/L, P = 0.16; 108.55 ± 4.37 versus 113.60 ± 5.71 mmol/L, P = 0.02). Nonsurvivors had significantly higher Dchloride levels (9.70 ± 4.62 versus 6.79 ± 3.59, P = 0.04). Lactate measurements and pH levels did not significantly differ between the groups (Table 2). The initial base deficit was similar between groups but the measurement of the 48-h base deficit was significantly higher in nonsurvivors (−4.80 ± 3.95 versus −6.56 ± 4.53, P = 0.08; −1.89 ± 1.51 versus 4.18 ± 1.79, P = 0.04). Counts of white blood cell and platelets, levels of hemoglobin, ALT, and AST, and the INR were similar between both groups. Finally, multivariate logistic regression analysis was used to evaluate the relationship between hyperchloremia and 28-day hospital mortality by calculating the 95% confidence interval (95%CI) and odds ratio (OR). For the multivariate analysis, possible factors identified by the bivariate analysis were included in the model to detect independent predictors of the outcome. In multivariate analysis, hyperchloremia at 48 h was shown to be an independent risk factor for in-hospital mortality (OR 1.8; 95%CI 1.05–2.1, P = 0.04) (Table 3). ISS and totally infused fluid volume at 48 h were also found to be independent risk factors for mortality (OR 1.85; 95%CI 1.02–2.69, P = 0.01; OR 1.43; 95%CI 0.97–2.03, P = 0.05, respectively).

**Table 1:** Baseline demographic and clinical variables of survivors and nonsurvivors

Variables		Survivors (n = 66)	Nonsurvivors (n = 20)	P value
Age (month), mean ± SD		91.32 ± 54.27	96.90 ± 55.49	0.77
Sex (female n /%, male n/%)		22 (40%); 44 (60%)	8 (33.3%); 12 (66.7%)	0.61
Respiratory rate (rate/min), mean ± SD		25.62 ± 4.60	24.60 ± 2.45	0.50
Peripheral oxygen saturation, %*		94.50 (5)	89.50 (5)	0.89
Heart rate (rate/min), mean ± SD		132.06 ± 23.15	134.7 ± 27.31	0.13
Mean arterial pressure (mmHg), mean ± SD		67.18 ± 12.43	64.10 ± 6.87	0.21
Type of trauma			
	Fall from height	34 (51.5%)	11 (55%)	0.95
	Traffic accident	32 (48.5%)	9 (45%)	
Duration of mechanical ventilation (day)*		4 (2)	5 (3)	0.80
Duration of PICU (day), mean ± SD		8.61 ± 2.60	5.90 ± 2.23	0.09
BSA, mean ± SD		0.96 ± 0.39	0.94 ± 0.40	0.89
GCS, mean ± SD		8.56 ± 3.12	7.3 ± 2.31	0.70
ISS*		16(3)	21(5)	0.01
Total infused fluid volume during 48 h (liter)		5.1 (2.8)	5.8 (3.7)	0.04

* Median (interquartile range).

PICU = pediatric intensive care unit; BSA = body surface area; GCS = Glasgow coma scale; ISS = injury severity score; SD = standard deviation.

**Table 2:** Comparison of laboratory findings between survivors and nonsurvivors

Variables		Survivors (n = 66)	Nonsurvivors (n = 20)	P value
Creatinine (mg/dL)				
	Initial, mean ± SD	0.46 ± 0.29	0.61 ± 0.45	0.23
	At 48 h, mean ± SD	0.48 ± 0.25	0.64 ± 0.25	0.07
Sodium (mmol/L)				
	Initial*	140 (3.25)	141.5 (2.75)	0.26
	At 48 h, mean ± SD	142.67 ± 2.62	149.90 ± 6.88	<0.01
Potassium (mmol/L)				
	Initial, mean ± SD	4.01 ± 2.89	4.13 ± 0.31	0.31
	At 48 h, mean ± SD	4.04 ± 0.26	3.95 ± 0.53	0.42
Chloride (mmol/L)				
	Initial, mean ± SD	103.76 ± 2.52	104.60 ± 2.83	0.16
	At 48 h, mean ± SD	108.55 ± 4.37	113.60 ± 5.71	0.02
Chloride (mmol/L)				
At 48 h				
	< 110	31 (77.5%)	3 (25%)	0.05
	≥ 110	9 (22.5%)	9 (75%)	
Δchloride (mmol/L), mean ± SD		6.79 ± 3.59	9.70 ± 4.62	0.04
pH				
	Initial*	7.35 (0.08)	7.33 (0.19)	0.14
	At 48 h, mean ± SD	7.40 ± 0.02	7.38 ± 0.03	0.10
Base deficit (mmol/L)				
	Initial, mean ± SD	-4.80 ± 3.95	-6.56 ± 4.53	0.08
	At 48 h, mean ± SD	-1.89 ± 1.51	-4.18 ± 1.79	0.04
Lactate (mmol/L)				
	Initial*	4.30 (1.6)	5.1 (4.3)	0.12
	At 48 h*	2.5 (1.3)	3.0 (2.1)	0.10
Hgb gr/dl *		11.45 (2.68)	10.60 (4.08)	0.43
PLT × 10^3^/μL, mean ± SD		237 ± 112	167 ± 92	0.08
WBC × 10^3^/μL, mean ± SD		16.41 ± 5.37	16.74 ± 9.40	0.88
ALT (U/L)*		128 (27)	132 (258)	0.42
AST (U/L)*		137 (66)	148 (51)	0.36
Albumin g/dL, mean ± SD		3.71 ± 0.58	3.40 ± 0.38	0.11
INR (U/L), mean ± SD		1.48 ± 0.37	1.71 ± 0.27	0.07

* Median (interquartile range).

Hb = Hemoglobin; WBC = white blood cell; PLT = platelet; INR = international normalized ratio; AST = aspartate aminotransferase; ALT = alanine aminotransferase; SD = standard deviation.

**Table 3:** Multivariate logistic regression analysis of survivors versus nonsurvivors

Variables	Odds Ratio	95% Confidence Interval	P value
ISS	1.85	1.02–2.69	0.01
Total infused fluid volume during 48 h (liter)	1.43	0.97–2.03	0.05
Chloride at 48 h	1.8	1.05–2.1	0.04
Δchloride	0.95	0.84–1.08	0.07

ISS = Injury severity score; Δchloride = delta chloride.

## DISCUSSION

The current study was a retrospective clinical study that evaluated the correlation between hyperchloremia and hospital mortality of seriously injured pediatric patients. The strengths of this research are the choice of a representative model of patients with major trauma admitted to the PICU and the performance of multivariate analysis for clinical confounders such as total infused fluid volume, base deficit, and injury severity score that are directly associated with hyperchloremia and mortality,

The main outcomes of this study were as follows: First, all patients had normal serum chloride levels on admission; however, hyperchloremia occurred more commonly in nonsurvivors after the initiation of medical therapy. Second, hyperchloremia, ISS, and the total volume of fluid infused in the first 48 h were found to be independently associated with mortality in children admitted to the PICU in multivariate logistic regression analysis.

The ISS score is frequently used to estimate the probability of mortality and survival ratio for patients with trauma.^10^ In a study comparing mortality scores in pediatric patients with major trauma, the ISS score was superior to the pediatric trauma score, pediatric risk of mortality III score, and base deficit, INR, and Glasgow coma scale score.^11^ The ISS scoring system has been shown to be better than the RTS in predicting mortality.^12^ In our study, although mortality scores were not compared to determine the accuracy of survival prediction, the ISS score was identified as an independent risk factor for mortality.

The most common cause of hyperchloremia after admission to the intensive care unit is an infusion of chloride-rich solutions, such as HTS and/or NS in patients with trauma.^13^ Normal saline is frequently used for the dilution of medications, and HTS is used for cerebral antiedema treatment, which may be the cause of the unnoticed origins of chloride in the PICU. The chloride concentration of normal saline is 154 Eq/L while the chloride concentration of 3% hypertonic saline is 513 Eq/L and is higher than the normal plasma chloride concentration.^14^ The very close chloride values among the groups in the present research may complicate the clinical applicability. However, the cutoff level of hyperchloremia was defined as a serum chloride level of > 110 mmol/L in this study. When the chloride levels of the two groups were compared at 48 h, 75% of the nonsurvivor group had a chloride value > 110 mmol/L compared with only 22.5% of the survivor group, which was a significant difference.

Many studies have shown that an increased mortality ratio might be associated with elevated serum chloride levels in patients with major trauma.^15,16^ Hyperchloremia can be harmful to tissues, causing an iatrogenic threat to the metabolic energy of cells and contributing to the risk of mortality and morbidity.^17^ However, avoiding excessive serum chloride overload is necessary. The pathophysiological mechanisms underlying the relationship between hyperchloremia and mortality from trauma have not yet been elucidated. Proinflammatory reactions are mediated by nitric oxide and have a higher ratio of interleukin (IL)-6 to IL-10 in lactic acidosis than in hyperchloremic metabolic acidosis.^18^ Serum chloride levels play a critical role in neutrophil function. Neutrophils need a permanent chloride influx to ensure sufficient is available substrate for hypochloric acid production,^19^ and an insufficient chloride concentration has been related to diminished neutrophil function.^20^ Although the cause of coagulopathy induced by isotonic saline is not exactly known, the administration of large volumes of isotonic saline may trigger coagulopathy.^21^ While fluid overload has been associated with morbidity and mortality in surgical patients, resuscitation volumes < 1,500 mL are not associated with increased mortality.^6,22^ Our study shows that nonsurvivors had a significantly larger volume of infused fluid than survivors. An increased risk of mortality was associated with an increase in the amount of fluid used for resuscitation, which may be a risk associated with using chloride-rich fluids for resuscitation. The volume-adjusted chloride load could not be calculated because of missing blood product data on the transfusion amount. However, higher chloride levels in the nonsurvivor group may be associated with patients in this group receiving more resuscitation volumes.

Administration of a large volume of chloride-rich solutions may have harmful outcomes for kidneys.^23^ Several experimental studies have demonstrated that large intravenous chloride loads decline renal blood flow, glomerular filtration rate, and renal cortical tissue perfusion.^24^ Similarly, a previous study showed that hyperchloremia at 48 h was prominently associated with acute kidney injury and hospital mortality in an ICU.^25^ Moreover, the glomerular filtration rate and extended hyperchloremia at rather than the highest serum chloride value or the duration of hypertonic fluid infusion were related to the development of acute kidney injury in patients with traumatic brain injury.^26^ In the current study, the serum creatinine level of nonsurvivors was insignificantly higher than that of survivors.

Besides hyperchloremia at 48 h, Δchloride was associated with 28-day mortality in major trauma patients.^8^ Huang et al. presented that new-onset hyperchloremia and increases of every 5 mmol/L in Δchloride were related to the increased probability of mortality.^27^ Similar to this research, Δchloride was markedly different between survivors and nonsurvivors in our study. This confirms the critical practical effect of using chloride-rich fluids, which can increase serum chlorine levels.

The present study had several limitations. First, this study was performed at a single medical institute, and the relatively small patient count may have limited our ability to clearly illuminate the association between hyperchloremia and mortality. Second, we evaluated serum chloride values only at admission and 48 h after PICU admission, and not at other time points during the stay in the PICU. Third, the total volume and concentration of the fluid infused before admission to the intensive care unit could not be measured. Finally, a larger number of patients is required to achieve considerable power and reliability.

## CONCLUSION

This study contributes to the increasing number of studies demonstrating that hyperchloremia may have inconvenient effects, especially in patients with trauma. Attention has recently focused on understanding the effect of serum chloride abnormalities on unfavorable outcomes. This study shows that chloride disorders and unfavorable outcomes appear to be associated with the ICU. Therefore solutions containing electrolytes separate from physiological solutions should be used more appropriately, and the development of hyperchloremia should be considered as a prognostic marker, taking into account the severity of the patient.
